# Key Features of Engagement Strategies in Nutrition Apps for Adults: Scoping Review

**DOI:** 10.2196/82276

**Published:** 2026-05-12

**Authors:** Maria F Vasiloglou, Zoë van der Heijden, Eric Antoine Scuccimarra, Alberto Conde Freniche, Desiree A Lucassen, Nienke de Vlieger, Frédéric Ronga, Elske Brouwer-Brolsma, Tamara Bucher

**Affiliations:** 1 Nestlé Institute of Health Sciences, Nestlé Research Lausanne Switzerland; 2 Division of Human Nutrition and Health, Wageningen University & Research Wageningen The Netherlands; 3 School of Health Sciences, Faculty of Health and Medicine, University of Newcastle Callaghan, NSW Australia; 4 Strategic Research Operations Unit, Nestlé Research Lausanne Switzerland; 5 Division of Nutrition and Dietetics, Bern University of Applied Sciences Bern Switzerland

**Keywords:** mobile health, mHealth, nutrition apps, user engagement, digital health, behavior change, scoping review, mobile apps, dietary behavior

## Abstract

**Background:**

Nutrition apps offer scalable opportunities to support dietary behavior change and prevent chronic diseases. Their success depends on sustained user engagement, which is essential yet challenging to achieve and, consequently impacts the long-term effectiveness of these digital tools. Engagement strategies have been widely explored in digital health, but a comprehensive synthesis focusing on nutrition apps for adults is lacking.

**Objective:**

This scoping review aimed to map the current engagement approaches and metrics implemented in nutrition apps targeting adults and to identify how user engagement is defined across studies.

**Methods:**

We conducted a search of the PubMed, Scopus, Cochrane, and Web of Science databases for relevant studies published from January 1, 2013, to June 30, 2024. The inclusion criteria included original adult interventional or observational studies that evaluated nutrition apps and reported user‑engagement strategies or metrics. Two reviewers independently screened records in Covidence, with discrepancies resolved by a third reviewer. Data were charted across study characteristics, engagement strategies, and engagement metrics and then synthesized narratively.

**Results:**

A total of 59 studies that used apps to improve dietary behaviors were included in our analysis, including randomized controlled trials, observational trials, and mixed methods studies. Most of these apps were designed for adults who were overweight and obese. The studies were primarily conducted in North America and Europe and were randomized controlled trials or nonrandomized intervention studies, with varying durations and sample sizes. Engagement strategies varied widely, and engagement was typically measured by frequency of specific function use and frequency of app use, followed by retention rate. The most common engagement strategies reported in studies were push notifications (n=29, 49%), behavioral theory integration (n=24, 41%), personalization and customization (n=19, 32%), and goal‑setting features (n=18, 31%). Only 31% (n=18) of studies provided an explicit definition of “user engagement,” and definitions were highly heterogeneous. Engagement measurement was dominated by quantitative system‑recorded metrics, including time and frequency of using specific functions (n=38, 64%), app use frequency (n=34, 58%), and retention (n=17, 29%). Few studies assessed qualitative or long‑term engagement dimensions, and long‑duration studies rarely integrated adaptive or contextualized engagement mechanisms. Research apps more frequently used theory‑driven strategies compared with commercial apps, which tended to emphasize streamlined user experience.

**Conclusions:**

Although several engagement strategies are commonly used, their implementation is inconsistent and often lacks grounding in conceptual frameworks. Research in the future needs to prioritize the use of common definitions for user engagement and measurement criteria while implementing user-centered design methods and using multiple research approaches to study the complex patterns of user engagement. The evidence base for engagement strategies needs strengthening because it will support the development of sustainable nutrition mobile health interventions.

## Introduction

### Background

Noncommunicable diseases, including cardiovascular disease, cancer, respiratory conditions, and diabetes, are the leading causes of death globally, accounting for more than 70% of deaths worldwide [1]. Overweight and obesity significantly increase the risk of these noncommunicable diseases, impacting morbidity and mortality rates [1]. The global obesity epidemic is worsening, with significant implications for physical and economic health [2].

Digital health tools for diet monitoring, planning, and precision nutrition have the potential to provide scalable tools to support health care professionals in managing nutrition-related diseases and obesity [3]. Despite the potential benefits, the integration of digital health tools into large, complex health systems remains limited. Challenges such as the need for user-centered, context-dependent, and customizable designs hinder widespread adoption, particularly in low-income countries with limited financial resources [4].

Mobile health (mHealth) apps, specifically those targeting nutrition, have proliferated over the past decade, aiding users in tracking food intake, providing personalized dietary advice, and promoting healthier lifestyles. Τhere were more than 330,000 digital health apps available, with a substantial proportion focusing on nutrition [5]. These apps are increasingly being recognized for their potential to improve poor dietary habits and diet-related chronic diseases. The scalability and wide reach of mHealth apps, combined with their capacity to deliver personalized dietary guidance at a low cost, position them as a valuable approach to promote healthier diets and thereby support public health efforts to reduce the burden of chronic disease [6]. Two primary categories can be identified based on the developers’ affiliations and goals: academic apps are created by nutrition or dietetics experts to offer a dependable, scientifically validated tool, primarily for research purposes. Conversely, consumer apps are usually developed by private companies specializing in digital development, aimed at the general public, with a primary focus on commercial objectives [7].

Although there is modest evidence that nutrition apps can be effective in promoting healthier dietary behaviors [6], a significant challenge lies in maintaining long-term user engagement with the app (“Little e”), which is necessary to ensure sufficient exposure to relevant behavior change techniques [8] and ultimately engagement in the behavior change process and outcomes (“Big E”) [9].

Although there is modest evidence that nutrition apps can be effective in promoting healthier dietary behaviors [6], a significant challenge lies in maintaining long-term user engagement, which is necessary to ensure sufficient exposure to relevant behavior change techniques [8]. User engagement is a multifaceted concept and includes both the extent of app use, such as frequency, duration, and depth of access, as well as the quality of the user experience, including attention, interest, and emotional involvement. Despite the growing popularity of mHealth apps, many nutrition apps struggle to retain user engagement over time [10-12]. For example, users of the commercial weight loss app Lose It! engaged for an average of only 29 days [13]. Similarly, in a short-term intervention, participants recorded vegetable intake on just 11 out of 28 days, with engagement declining as the study progressed [14]. Low engagement is a common challenge in app-based interventions, with approximately 71% of users discontinuing within the first 90 days. Since this significantly undermines effectiveness [15-17], it is crucial to understand the factors that support sustained user engagement [18].

In an effort to understand user engagement in mHealth apps, several systematic reviews have been conducted to synthesize the different approaches in digital health interventions, particularly in mental health [19-23], physical activity, and chronic disease prevention and management [18,24,25]. In addition to these focused reviews, broader systematic reviews have also been performed, encompassing digital health interventions through multiple modes of delivery beyond mHealth apps, combining a variety of lifestyle behaviors [26,27]. Furthermore, specific reviews have examined digital health interventions for population groups, such as children [28] or older adults [29]. These studies provide an overview of useful engagement strategies in mHealth more broadly and for specific applications (ie, physical activity, mental health, and chronic disease).

To address the challenge of sustaining engagement in nutrition apps, various strategies have been implemented to enhance motivation and user experience, including gamification, personalization [30], and the integration of social features [31]. However, to the best of our knowledge, no review has yet provided a comprehensive overview of the full range of strategies used to promote user engagement in nutrition apps for the general adult population.

### Objectives

This scoping review aims to provide an overview of the current landscape of predefined strategies implemented in apps used for nutrition interventions to enhance user engagement, identify gaps in the literature, and guide future research and app development. Another goal is to map and synthesize the definitions of user engagement used in the studies, creating a comprehensive and unified definition.

## Methods

### Overview

We chose a scoping review to explore how user engagement is defined, measured, and promoted in nutrition apps due to the broad and heterogeneous nature of the literature across disciplines such as nutrition, behavioral science, and digital health. This approach enables a comprehensive overview of existing evidence without limiting study design or outcomes. This review is guided by the methodological framework originally proposed by Arksey and O’Malley [32], which outlines 5 key stages: identifying the research question, identifying relevant studies, study selection, charting the data, and summarizing and reporting the results. This scoping review was conducted in accordance with the PRISMA-ScR (Preferred Reporting Items for Systematic Reviews and Meta-Analyses extension for Scoping Reviews) checklist, which can be found in Multimedia Appendix 1.

### Search Strategy and Selection Criteria

We searched 4 databases, namely PubMed, Scopus, Cochrane, and Web of Science, for relevant studies published between January 1, 2013, and June 30, 2024. The detailed search strategy can be found in Multimedia Appendix 1.

### Eligibility Criteria

Our inclusion and exclusion criteria followed the population, intervention, comparison, outcome, and study design framework (Multimedia Appendix 1). This scoping review included studies that examined engagement strategies within nutrition apps used by adults aged above 18 years, across a range of health conditions. The review focused on interventions that incorporate elements such as gamification, personalized content, social integration, and behavior change techniques aimed at enhancing user engagement. Excluded were studies involving children and adolescents below 18 years, and those focusing on non–nutrition-related apps (eg, fitness-only or medication adherence apps without a primary nutrition component). Furthermore, studies that assessed engagement without reporting specific metrics such as daily active users, time spent in the app, or adherence rates were excluded. Only original interventional and observational studies were considered, excluding any type of review (eg, umbrella reviews, scoping reviews, or narrative reviews); conference abstracts; case reports; expert opinions without empirical data; and editorials, as well as quality and content analyses of apps found in app stores.

### Screening and Data Extraction

Covidence software was used to manage the screening flow, beginning with the removal of duplicate records, followed by title and abstract screening conducted by pairs of reviewers (MFV, EAS, EB-B, TB, ACF, FR, SL, NvD, and ZvdH). All potentially eligible abstracts underwent full-text assessment by pairs of reviewers (MFV, EAS, SL, ACF, EB-B, FR, ZvdH, NdV, and DAL). Data extraction of eligible papers was performed by a single investigator and then verified by a second investigator (MFV or ZvdH). Any discrepancies were resolved through discussion with a third reviewer (EB-B).

An Excel (Microsoft Corp) data extraction spreadsheet was developed to systematically compile the following study-level information: author, year, aim, study design, location, total number of participants, female or male, intervention group, control group, duration, age, BMI, condition or disease, type of app, commercial or research, app features, intervention description, incentive, user-engagement strategies, description of those strategies, engagement definition, app evaluation criteria, specification of metrics, description of metrics, relationship between user engagement with smartphone apps and main outcome, app evaluation results, and strengths and limitations. Extracted data on engagement strategies were classified into the following categories: push notifications, personalization, goal setting, game-like features, multimedia formats, monitoring (real time or passive), peer-to-peer communication, access to professionals, content reliability, quality of feedback, behavioral theory, or none or insufficiently specified. User metrics (ie, engagement metric or indicators as specified in the protocol) were categorized into average session length, daily app use, drop-offs, most visited or active screens, overall time used, retention rate, time and frequency of using specific functions, frequency of app use, and others.

The effect on health outcomes were extracted from the included studies and were first categorized as follows: (1) *positive outcomes*—significant improvements (*P*<.05) or positive trends (*P*<.10), (2) *negative outcomes*—significant deterioration (*P*<.05) or negative trends (*P*<.10), (3) *neutral or no outcomes*—no significant changes (*P*≥.10), or (4) not reported—no or insufficient data on health outcomes. Subsequently, the number of studies reporting positive, neutral, or neutral or no outcomes was counted for each category of user metrics to describe how frequently each metric appeared in studies with different types of outcomes. This counting procedure was purely descriptive and was not intended to infer causality, comparative effectiveness, or the predictive value of specific engagement metrics.

Finally, the duration of the studies was defined as short when it lasted less than a month, medium when it lasted 1 to 3 months, and long when it lasted more than 3 months. These classifications were developed specifically for this study by the authors.

## Results

In the following section, the outcomes from the screening and analysis process are presented, including the characteristics of the included studies, the engagement strategies, and the metrics identified.

### Search Results

**Figure 1:**
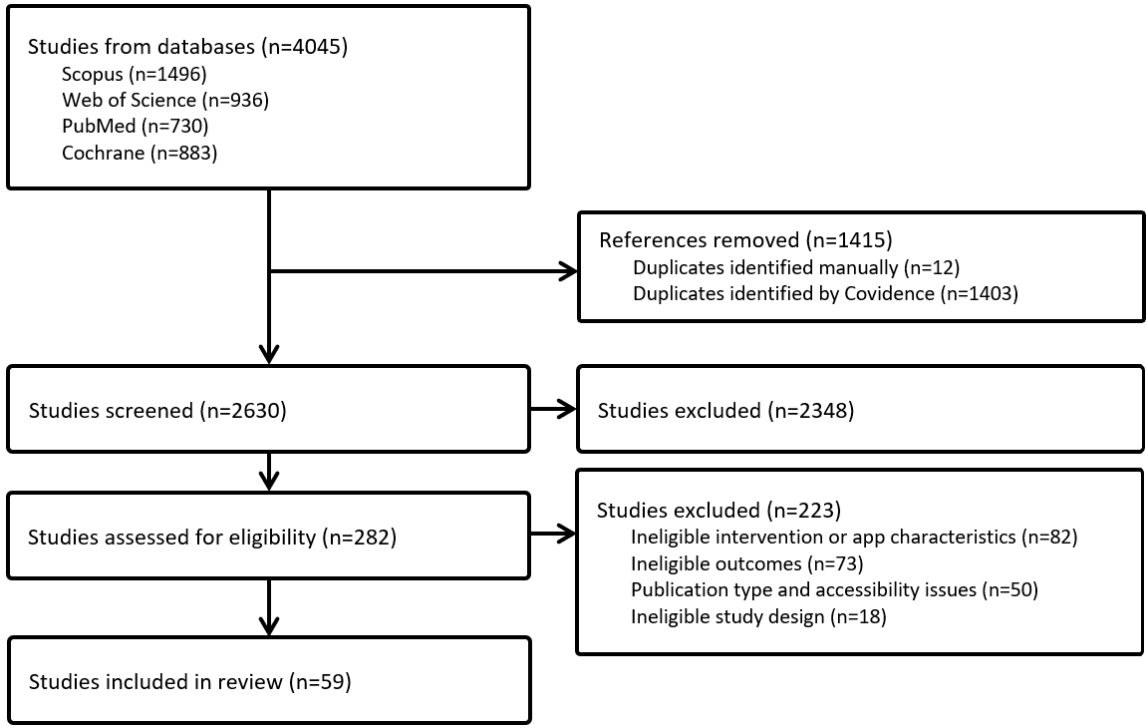
PRISMA-ScR (Preferred Reporting Items for Systematic Reviews and Meta-Analyses extension for Scoping Reviews) flowchart.

Our searches retrieved 4045 records, and after the exclusion of duplications, 2630 (65%) records underwent abstract screening, of which 282 (7%) underwent full-text review (Figure 1). On the basis of the established inclusion and exclusion criteria, 59 studies were included in our scoping review (Multimedia Appendix 1).

### Characteristics of Included Studies

#### Population

The scoping review included a total of 59 studies examining nutrition apps. The most common study design was a randomized controlled trial (RCT), accounting for 41% (24/59) of the studies. The remaining study designs were nonrandomized intervention studies (11/59, 19%), mixed methods studies (10/59, 17%), observational studies (8/59, 14%), and secondary analyses (6/59, 9%). Most studies (26/59, 44%) were conducted in North America, followed by Europe (14/59, 24%), Australia (9/59, 15%), Asia (7/59, 12%), and New Zealand (2/59, 3%); 1 (N=59, 2%) study did not specify a location. Sample sizes varied across studies from 12 to 1,011,008. Most of the participants (50/59, 85%) were adults (aged 18-55 years). In 24% (14/59) of the studies, women made up 75% to 90% of the participants, and in 39% (23/59) of the studies, women constituted 50% to 75% of the study population.

The duration of studies also varied widely. Most studies (22/59, 37%) had a medium duration of 1 to 3 months. A smaller proportion (10/59, 17%) had a short duration of less than 1 month, including 2 (N=59, 3%) studies that lasted 1 week or less. Studies with a long duration of more than 3 months accounted for 32% (19/59), spanning from over 3 months to over a year. A notable portion did not specify the study duration or left it unreported (8/59, 14%). Most studies (24/59, 41%) focused on weight management. Diabetes (type 1, type 2, and gestational) was the focus in 10% (6/59) of the studies, while pregnancy was the target in 7% (4/59) of the studies. Table 1 provides detailed information about the studies and their populations.

In our analysis, the distribution between commercial and research-based nutrition apps was assessed. Research apps constituted the majority, comprising 75% (44/59) of the studies included in our review. In contrast, commercial apps were represented in 25% (15/59) of the studies. Detailed characteristics of the included studies can be found in Multimedia Appendix 1.

**Table 1:** Basic characteristics of the study populations (N=59).

Characteristic			Studies, n (%)
**Participants**			
	≤20	6 (10)	
	20-50	9 (15)	
	50-100	14 (24)	
	100-200	14 (24)	
	200-500	7 (12)	
	500-1000	3 (5)	
	>1000	6 (10)	
**Duration**			
	≤1 month	10 (17)	
	1 month-3 months	22 (37)	
	≥3 months	19 (32)	
	Not specified or not applicable	8 (14)	
**Mean age group (y)**			
	18-65	53 (90)	
	≥65	3 (5)	
	Not reported	3 (5)	
**Mean BMI (kg/m^2^**)			
	Underweight (<18.5)	0 (0)	
	Normal (18.5-24.9)	4 (7)	
	Overweight (25.0-29.9)	14 (24)	
	Obesity 1 (30.0-34.9)	19 (32)	
	Obesity 2 (35.0-39.9)	6 (10)	
	Not reported	20 (34)	
**Condition or disease**			
	Overweight or obesity	24 (41)	
	Diabetes (type 1, type 2, or gestational)	6 (10)	
	Pregnancy	4 (7)	
	Mental health conditions	3 (5)	
	Older adults	2 (3)	
	Suboptimal lifestyle habits	2 (3)	
	Others	7 (14)	
	Not reported	12 (20)	

#### Features

A variety of user-engagement strategies were used across the included nutrition apps (refer to Figure S1 in Multimedia Appendix 1). The most frequently applied strategies were push notifications or prompts (29/59, 49%), behavioral theory (24/59, 41%), personalization and customization (19/59, 32%), and goal setting (18/59, 31%). Less commonly reported were features such as peer-to-peer communication (9/59, 15%), game-like features (8/59, 14%), and multimedia formats (5/59, 8%). A total of 4 (N=59, 7%) studies did not report on user-engagement strategies applied or described them insufficiently.

To better understand how these strategies were operationalized, data were extracted on the specific app features used. The most common feature was self-monitoring or tracking (44/59, 75%), followed by prompts or notifications (25/59, 41%). Personalized feedback (22/59, 37%), goal-setting tools (15/59, 27%), and outcome tracking or progress summaries (13/59, 22%) were also frequently included. Less commonly used features included multimedia materials and social features (both 11/59, 19%), data sharing or professional access (9/59, 15%), and more advanced technologies such as artificial intelligence chatbots or image recognition (2/59, 3%). A small number of studies reported features such as rewards (4/59, 7%), reflective logging tools (7/59, 12%), or photo-based modules (6/59, 10%).

#### User-Engagement Metrics Included

To evaluate how users interacted with these features, most of the studies (38/59, 64%) included metrics of functions related to time and frequency of specific functions. Many of them also included frequency of app use (34/59, 58%). Moreover, some of the studies (17/59, 29%) included retention rate and others included the number of drop-offs (15/59, 25%). Fewer studies (n=11, 19%) included metrics such as the overall time that an app was used, the daily app use time (9/59, 15%), the most visited or the most active screens (8/59, 14%), and the average session length (3/59, 5%; Figure S2 in Multimedia Appendix 1).

Table 2 presents the health outcomes reported in studies that included a given engagement metric. For each metric, we examined only the subset of studies that reported that specific metric and assessed whether they described improvements, no changes, or insufficient detail regarding effects on health outcomes. Most metrics were included in studies reporting positive effects. There were no studies that reported a negative association of a metric with health outcomes or components.

**Table 2:** Frequency of engagement metrics applied, used, and corresponding health outcomes reported in studies that included the specific metric (N=59).

Engagement metric	Improvements or positive changes, n	No changes, n	Not reported or insufficient detail, n	Studies, n (%)
Time or frequency of using specific functions	14	5	19	38 (64)
Frequency of app use	12	6	16	34 (58)
Retention	6	2	9	17 (29)
Drop-offs	2	3	10	15 (25)
Overall time used	4	3	4	11 (19)
Retention	3	0	7	10 (17)
Daily app use	4	1	4	9 (15)
Most visited or active screens	2	1	5	8 (14)

#### Commercial Versus Research Apps

A total of 15 (N=59, 25%) commercial apps and 44 (75%) research apps were analyzed to compare user-engagement strategies. Push notifications or prompts were used in 33% (5/15) of commercial apps and in 55% (24/44) of research apps, suggesting that research apps may more frequently incorporate reminder-based engagement. Personalization and customization features were equally present in commercial and research apps, each used by 33% (5/15) and 32% (14/44) of the apps, respectively. Goal setting was more common in research apps (15/44, 34%) compared to commercial apps (3/15, 20%). Game-like features were used by 13% (2/15) of commercial apps and 14% (6/44) of research apps.

#### App Evaluation Results Among Apps That Included Evaluation

In our review, 32 (54%) of the 59 included studies reported various dimensions of app evaluation. The most frequently assessed aspect was user satisfaction, noted in 50% (16/32) of the studies that included evaluations. This was followed by ease of use, reported in 25% (8/32) of the studies. App functionalities were evaluated in 28% (9/32) of the studies. Aesthetics, intention to use, and attitude toward the app were each evaluated in 9 (n=32, 28%) studies. Attitude toward one’s own health was assessed in only 3% (1/32) of the studies. Suggestions for improvement were noted in 19% (6/32) of the studies. Additionally, 19% (6/32) of the studies did not report specific categories of user feedback.

#### App Evaluation Criteria

Feasibility was the most frequently assessed criterion, reported in 13 of 59 (22%) studies. This was closely followed by user satisfaction, which was evaluated in 12 of 59 (20%) studies. Usability and acceptability were each assessed in 10 of 59 (17%) studies. Finally, 26 of 59 (44%) studies did not report any specific evaluation criteria.

#### Key Engagement Strategies Among Adult Populations With Specific Needs

Key approaches varied across the 30 studies analyzing engagement strategies in apps targeting specific populations of adults. Among the 24 studies on apps for individuals with overweight and obesity, push notifications and prompts (n=12, 50%), behavioral theory (n=12, 50%), goal setting (n=8, 33%), peer-to-peer communication (n=5, 21%), and access to professionals (n=5, 21%) appeared as key engagement strategies. Engagement tracking focused on assessing the time and frequency of using specific functions (15/24, 63%), app use frequency (15/24, 63%), and the retention rate (11/24, 46%). Similarly, the 6 studies on apps for individuals living with diabetes prioritized behavioral theory (n=5, 83%), with half (n=3, 50%) of them incorporating push notifications, professional support, and real-time monitoring features, while goal setting (n=1, 17%) and passive data monitoring (n=2, 33%) were less common.

#### Variation in Engagement Strategies Across Study Durations

In studies with different durations, distinct patterns emerged. Of the 10 (17%) short-duration studies (<1 month), 8 (80%) studies used push notifications or prompts extensively. The 22 (N=59, 37%) medium-duration studies (1-3 months) highlighted the importance of behavioral theory, with 11 (50%) of them incorporating it. The 19 (N=59, 32%) long-duration studies (>3 months) also favored behavioral theory and push notifications equally, each used in 42% (8/19) of the cases.

Short-duration studies prioritized tracking the time and frequency of using specific functions (7/10, 70%) and the frequency of app use (6/10, 60%). In medium-duration studies, 73% (16/22) focused on the time and frequency of using specific functions, and 55% (12/22) focused on the frequency of app use. Long-duration studies showed a significant measurement of time and frequency of using specific functions (9/19, 47%) and retention rates (6/19, 32%).

The presence of defined engagement strategies was examined across 59 studies. Of these, 18 (31%) studies provided an explicit definition of user engagement, while 9 (15%) studies presented an implicit conceptual definition. However, most of the studies (32/59, 55%) did not define user engagement at all. Among the 27 studies that contained either explicit or implicit definitions, various dimensions were highlighted. The most referenced criterion was use patterns, mentioned in 21 (n=27, 78%) studies. Feature interaction was the second most common, appearing in 8 (n=27, 30%) studies, followed by behavioral indicators in 6 (n=27, 22%) studies and user feedback and responses in 5 (n=27, 19%) studies. Outcome-based definitions, linking engagement to specific user achievements or health outcomes, were noted in 3 (n=27, 11%) studies. The least common approach was game-like features, mentioned in just 1 (n=27, 4%) study.

To summarize how user engagement was operationalized across the included studies, we derived a working description based on those studies that provided an explicit definition of user engagement (18/59, 31%). This description is intended as a synthesis of observed operationalizations rather than a novel or normative definition:

User engagement is operationalized as the extent, frequency, and persistence of users’ interactions with the app over time. It’s typically quantified using system-recorded metrics, such as the number of active or logged-in days, total time spent in the app, frequency of accessing app modules, and the number or intensity of recorded actions (e.g., logging meals or viewing content). In some cases, these indicators are combined into composite scores, points totals, or weekly usage summaries, while other studies operationalize express engagement as the proportion of days with recorded activity. A small number of studies additionally incorporate the user’s reflections on factors that support or hinder continued app usage.

## Discussion

### Principal Findings

This scoping review provides a comprehensive overview of 59 mHealth nutrition studies, focusing on user-engagement strategies and metrics. Most apps targeted adults with overweight and/or obesity and were tested in RCTs and nonrandomized intervention studies. The included study samples comprised a predominantly female population. Study durations and sample sizes varied. Engagement was typically measured by the time and frequency of using specific functions and overall app use frequency. Push notifications and behavioral theory elements were the most frequently reported engagement strategies.

Push notifications and behavioral theory (elements) were commonly used to enhance engagement, aligning with findings from other digital health interventions, including mental and social well-being [33], diabetes management [34], weight loss interventions [35], or physical activity promotion [36]. While their widespread use suggests versatility across health contexts and adult populations, poorly timed or excessive push notifications can lead to user fatigue or disengagement, emphasizing the need to optimize the implementation of the engagement strategies. Notably, user engagement is known to be influenced by individual factors, including an individual’s digital literacy and technology acceptance [37,38], health and demographic characteristics [39], or behavioral and psychological factors [40]. In addition, contextual factors also affect user engagement. For instance, sending notifications at specific times during weekends may yield better engagement rates [41,42]. Therefore, a standardized approach may not engage all users effectively, highlighting the need for adaptive frameworks that tailor engagement strategies to individual user characteristics and contextual factors.

Looking into the differences between commercial and research apps, the latter often used structured engagement tools such as push notifications and goal setting, likely reflecting a focus on behavioral theory and evidence-based practices. In contrast, commercial apps tended to emphasize user experience and simplicity since they are designed for broader appeal, with minimal features to reduce user burden. Both app types similarly adopted personalization and game-like elements, suggesting their broad value. For future studies and development, commercial apps could adopt more evidence-based strategies, while research apps might benefit from commercial design strengths.

The adoption of evidence-based strategies in mHealth apps is essential to ensure their effectiveness, equity, and sustainability in real-world settings. Evidence-based approaches integrate the best available scientific evidence with professional expertise and contextual considerations, thereby reducing reliance on ad hoc or intuition-driven decision-making [43]. In this review, engagement was primarily assessed using quantitative metrics, often referred to as “Little e” engagement, such as time spent on specific functions and overall app use, aligning with existing literature [25]. While standardized quantitative metrics aid comparability and benchmarking, they provide only a limited view on user engagement, which also involves emotional, cognitive, and behavioral dimensions [44]. Most studies focused on short-term metrics, limiting insights into sustained engagement. Therefore, longitudinal assessments are needed to better understand long-term user interaction, while qualitative measures, such as interviews and in-app feedback, could provide deeper insights into user experience motivations, satisfaction, perceived usability, and barriers, and for explaining why certain engagement strategies succeed or fail [44-46]. Therefore, a mixed methods approach may better capture engagement dynamics and may help optimize future mHealth nutrition tools to better meet user needs and preferences. This variation between research and commercial apps underscores the need for standardized guidelines to ensure consistent quality and effectiveness in behavior change apps, an aim already addressed by several ongoing initiatives [47].

This review highlights gaps in how user engagement is conceptualized, defined, and measured. Only 31% (n=18) of studies explicitly defined engagement, limiting comparability and generalizability, an issue noted in multiple previous reviews on the topic [48,49]. Despite existing conceptual frameworks [44,45], their adoption remains limited, leading to high heterogeneity in engagement metrics [50]. This inconsistency hinders the understanding of real-world challenges, such as low uptake of nutrition apps, and impedes the development of standardized measures. These measures are crucial for comparability, establishing benchmarks, and deriving robust conclusions on effective engagement strategies. Future research should prioritize implementing established frameworks and developing standardized metrics to enhance cross-study synthesis and support evidence-based best practices for mHealth nutrition tools.

### Limitations

Despite the broad scope of this review, several limitations must be acknowledged. The diversity of the included studies, both in design and in the involved engagement strategies, made direct comparisons of effectiveness challenging. Additionally, the findings highlight the need for further investigation into how engagement strategies can be tailored to individual user characteristics and contextual factors. The included studies use a wide range of methodologies, including RCTs, pilot RCTs, secondary analyses of RCTs, observational cohort studies, and mixed methods research, such as feasibility and acceptability studies. There is also significant variability in sample sizes, ranging from as few as 12 participants in the study by Robinson et al [51] to more than a million (1,011,008) in the study by Serrano et al [13]. Moreover, while this review analyses engagement among users who accessed the included interventions, it is crucial to acknowledge that nonuse may reflect structural and contextual barriers rather than a lack of interest. Several barriers, including limited access to digital infrastructure, low digital literacy, and socioeconomic constraints, may prevent certain populations from engaging with nutrition apps. The latter could affect both the extent and quality of recorded engagement, potentially leading to underestimation or misrepresentation of true user behavior. Thus, it is important to note that our findings represent the experiences of the included active users.

A significant limitation observed across the reviewed studies is the lack of clear, standardized definitions for essential terms such as “engagement,” which is often undefined, as well as broad concepts such as “acceptability” and “feasibility.” Furthermore, few studies include reflective discussions on how to improve user engagement, despite its critical role in the success of interventions [44]. The absence of standardized reporting metrics further complicates the synthesis of findings, limiting the ability to draw meaningful comparisons across studies. It should be noted that the counting of studies reporting positive, negative, or neutral findings across engagement‑metric categories was purely descriptive. This approach does not allow any causal, comparative, or predictive inferences regarding the effectiveness of specific engagement metrics, and the observed distribution reflects reporting patterns in the included studies rather than true differences in impact.

Finally, another limitation of this study was the uneven distribution between commercial and research-based nutrition apps across the reviewed articles, with the latter constituting a majority (n=44, 75%). Whereas commercial apps typically prioritize consumer design principles and advanced user interface aesthetics, research apps may focus more on evidence‐based content, and, in some cases, compensate users to sustain use. Therefore, the insights gained from this review can be further explored in future studies that incorporate a broader range of commercial apps.

### Future Directions

Future research should focus on more rigorous evaluations, potentially through RCTs or long-term observational studies, to establish clearer insights into the efficacy of these strategies.

Co-design approaches in the development of nutrition and health apps have been shown to enhance user engagement and improve intervention effectiveness. Many studies have used participatory methods, involving stakeholders in the design process to ensure that the app aligns with user needs and preferences. Some commonly used co-design techniques include participatory research approaches, stakeholder engagement, and iterative design workshops. According to Davis et al [52] and Jackson et al [53], the benefits of co-design include higher user satisfaction, improved app usability, and a greater likelihood of meeting users’ needs, particularly by addressing cultural considerations. While co-design in mHealth is still an emerging field, it has demonstrated effectiveness across various health domains, including disease management and health promotion. Further research is needed to fully implement co-design processes and evaluate their impact on nutrition interventions.

Although many studies report use patterns, such as log in frequency and session duration, few provide insights into how users engage with specific behavioral components of nutrition apps. This lack of detail makes it difficult to identify which features drive engagement and behavior change. Additionally, engagement metrics vary widely across studies, making comparisons and synthesis challenging. For example, “active use” may be defined as a single session in one study but as daily interactions in another, complicating efforts to draw meaningful conclusions.

Moreover, most studies rely on static, one-size-fits-all engagement metrics that do not account for changes in user behavior over time. Without adaptive metrics, where use patterns dynamically inform engagement strategies, apps risk missing opportunities for personalization and sustained engagement. This static approach limits the potential for iterative improvements and real-time feedback loops that could optimize user experience and long-term outcomes. A recent methodological framework has been developed to analyze and model the dynamic processes involved in participant engagement with mHealth apps and identify factors that foster or undermine engagement in an individual's respective context [54,55]. However, the uptake of this type of approach is still very limited. Future research on user engagement may find this type of approach particularly valuable to understand how different contextual factors impact engagement as a dynamic process.

### Conclusions

This review provides a broad overview of the various strategies used to enhance user engagement in nutrition apps. mHealth nutrition apps predominantly target adults with overweight or obesity and are often evaluated through controlled trials with female-majority samples. Despite variability in study design, engagement is commonly tracked via use patterns, with push notifications and behavioral theory–based strategies emerging as key tools to enhance user involvement. By understanding and implementing these strategies, we can design more engaging and effective digital tools to promote healthy dietary behaviors and ultimately contribute to improved public health outcomes.

## Appendix

Multimedia Appendix 1 Search strategy, PICOS, user-engagement definitions, list of included studies, basic study characteristics, and characteristics of the apps.

Multimedia Appendix 2 Preferred Reporting Items for Systematic Reviews and Meta-Analyses extension for Scoping Reviews (PRISMA-ScR) checklist.

## Abbreviations

mHealth: mobile health
PRISMA-ScR: Preferred Reporting Items for Systematic Reviews and Meta-Analyses extension for Scoping Reviews
RCT: randomized controlled trial

## Data Availability

The datasets generated or analyzed during this study are available from the corresponding author on reasonable request.
