# Supplementary material for: Key Features of Engagement Strategies in Nutrition Apps for Adults: Scoping Review
Source: JMIR Mhealth Uhealth. 2026 May 12;14:e82276. doi: 10.2196/82276 (PMC13213328; doi:10.2196/82276)

**APPENDIX**

1. **Search Strategy**

("mobile app*") OR ("smartphone app*")

**AND**

((nutrition OR diet* OR food OR "calorie tracking" OR "meal tracking" OR meal OR “weight loss” OR “weight control” OR “weight management” OR “food diary” OR “food log” OR “food intake” OR “dietary intake”)

**AND**

("engagement" OR "adoption" OR "retention" OR "behavior change" OR "gamification" OR "personalization" OR "motivation" OR "user experience” OR “attrition " OR “adherence” OR “drop out” OR “drop-out” OR “rewards” OR “incentive*”))

**Scopus 1496**

( TITLE-ABS-KEY ( "mobile app*" ) OR TITLE-ABS-KEY ( "smartphone app*" ) )

( TITLE-ABS-KEY ( nutrition ) OR TITLE-ABS-KEY ( diet* ) OR TITLE-ABS-KEY ( food ) OR TITLE-ABS-KEY ( "calorie tracking" ) OR TITLE-ABS-KEY ( "meal tracking" ) OR TITLE-ABS-KEY ( meal ) OR TITLE-ABS-KEY ( "weight loss" ) OR TITLE-ABS-KEY ( "weight control" ) OR TITLE-ABS-KEY ( "weight management" ) OR TITLE-ABS-KEY ( "food diary" ) OR TITLE-ABS-KEY ( "food log" ) OR TITLE-ABS-KEY ( "food intake" ) OR TITLE-ABS-KEY ( "dietary intake" ) )

( TITLE-ABS-KEY ( engagement ) OR TITLE-ABS-KEY ( adoption ) OR TITLE-ABS-KEY ( retention ) OR TITLE-ABS-KEY ( "behavior change" ) OR TITLE-ABS-KEY ( gamification ) OR TITLE-ABS-KEY ( personalization ) OR TITLE-ABS-KEY ( motivation ) OR TITLE-ABS-KEY ( "user experience" ) OR TITLE-ABS-KEY ( attrition ) OR TITLE-ABS-KEY ( "food diary" ) OR TITLE-ABS-KEY ( adherence ) OR TITLE-ABS-KEY ( "drop out" ) OR TITLE-ABS-KEY ( "drop-out" ) OR TITLE-ABS-KEY ( rewards ) OR TITLE-ABS-KEY ( incentive* ) )

( ( TITLE-ABS-KEY ( "mobile app*" ) OR TITLE-ABS-KEY ( "smartphone app*" ) ) ) AND ( ( TITLE-ABS-KEY ( engagement ) OR TITLE-ABS-KEY ( adoption ) OR TITLE-ABS-KEY ( retention ) OR TITLE-ABS-KEY ( "behavior change" ) OR TITLE-ABS-KEY ( gamification ) OR TITLE-ABS-KEY ( personalization ) OR TITLE-ABS-KEY ( motivation ) OR TITLE-ABS-KEY ( "user experience" ) OR TITLE-ABS-KEY ( attrition ) OR TITLE-ABS-KEY ( "food diary" ) OR TITLE-ABS-KEY ( adherence ) OR TITLE-ABS-KEY ( "drop out" ) OR TITLE-ABS-KEY ( "drop-out" ) OR TITLE-ABS-KEY ( rewards ) OR TITLE-ABS-KEY ( incentive* ) ) ) AND ( ( TITLE-ABS-KEY ( nutrition ) OR TITLE-ABS-KEY ( diet* ) OR TITLE-ABS-KEY ( food ) OR TITLE-ABS-KEY ( "calorie tracking" ) OR TITLE-ABS-KEY ( "meal tracking" ) OR TITLE-ABS-KEY ( meal ) OR TITLE-ABS-KEY ( "weight loss" ) OR TITLE-ABS-KEY ( "weight control" ) OR TITLE-ABS-KEY ( "weight management" ) OR TITLE-ABS-KEY ( "food diary" ) OR TITLE-ABS-KEY ( "food log" ) OR TITLE-ABS-KEY ( "food intake" ) OR TITLE-ABS-KEY ( "dietary intake" ) ) )

**COCHRANE 883**

(nutrition) OR (diet*) OR (food) OR (calorie NEXT tracking) OR (meal NEXT tracking) OR (meal) OR (weight NEXT loss) OR (weight NEXT control) OR (weight NEXT management) OR (food NEXT diary) OR (food NEXT log) OR (food NEXT intake) OR (dietary NEXT intake)

(engagement) OR (adoption) OR (retention) OR (behavior NEXT change) OR (gamification) OR (personalization) OR (motivation) OR (user NEXT experience) OR (attrition) OR (adherence) OR (drop NEXT out) OR (drop-out) OR (rewards) OR (incentive*)

(mobile NEXT app*):ti,ab,kw OR (smartphone NEXT app*):ti,ab,kw

**Pubmed 665**

((("mobile app*"[Title/Abstract]) OR ("smartphone app*"[Title/Abstract])) AND (((((((((((((("engagement"[Title/Abstract]) OR ("adoption"[Title/Abstract])) OR ("retention"[Title/Abstract])) OR ("behavior change"[Title/Abstract])) OR ("gamification"[Title/Abstract])) OR ("personalization"[Title/Abstract])) OR ("motivation"[Title/Abstract])) OR ("user experience"[Title/Abstract])) OR ("attrition"[Title/Abstract])) OR ("adherence"[Title/Abstract])) OR ("drop out"[Title/Abstract])) OR ("drop-out"[Title/Abstract])) OR ("rewards"[Title/Abstract])) OR ("incentive"[Title/Abstract]))) AND (((((((((((((nutrition[Title/Abstract]) OR (diet*[Title/Abstract])) OR (food[Title/Abstract])) OR ("calorie tracking"[Title/Abstract])) OR (meal tracking[Title/Abstract])) OR (meal[Title/Abstract])) OR ("weight loss"[Title/Abstract])) OR ("weight control"[Title/Abstract])) OR ("weight management"[Title/Abstract])) OR ("food diary"[Title/Abstract])) OR ("food log"[Title/Abstract])) OR ("food intake"[Title/Abstract])) OR ("dietary intake"[Title/Abstract]))

**WOS 936**

**engagement** (Topic) or **adoption** (Topic) or **retention** (Topic) or **"behavior change"** (Topic) or **personalization** (Topic) or **motivation** (Topic) or **"user experience"** (Topic) or **attrition** (Topic) or **adherence** (Topic) or **"drop out"** (Topic) or **"drop-out"** (Topic) or **rewards** (Topic) or **incentive** (Topic)

**nutrition** (Topic) or **diet*** (Topic) or **food** (Topic) or **"calorie tracking"** (Topic) or **"meal tracking"** (Topic) or **meal** (Topic) or **"weight loss"** (Topic) or **"weight control"** (Topic) or **"weight management"** (Topic) or **"food diary"** (Topic) or **"food log"** (Topic) or **"food intake"** (Topic) or **"dietary intake"** (Topic)

**"mobile app*"** (Topic) or **"smartphone app*"** (Topic)

1. **PICOS**

|  | **Inclusion** | **Exclusion** |
| --- | --- | --- |
| **Participants** | Adults aged >18y  Users of nutrition apps living with any condition or disease | Children and adolescents (<18 years old)  Studies focusing on populations not using nutrition apps (eg, users of fitness-only apps, medical devices, or non-nutritional health apps). Studies that do not specify the population or focus solely on healthcare providers or app developers. |
| **Intervention** | Strategies used to engage users in nutrition apps, which could include gamification elements, personalized content, social integration features, use of behavior change techniques. | Studies that examine engagement strategies for apps not focused on nutrition (eg, medication adherence apps, pure fitness apps, mental health apps). Studies that focus on hardware-based interventions like wearables without app integration. |
| **Comparator** | - | - |
| **Outcome** | Metrics or indicators of user engagement (eg, daily/weekly/monthly active users, time spent in-app, levels of interaction with app features, user ratings and reviews, compliance/adherence rates, etc.) | Studies that do not report any metrics or qualitative data on user engagement, adherence, or interaction with the nutrition app |
| **Setting** | Original intervention and observational studies | Umbrella reviews, Scoping reviews, Narrative reviews, Conference abstracts, case reports or expert opinions without empirical data, editorials, quality, and content analysis of apps found in app stores |

1. **User-engagement definitions**

| **Definition of engagement, if explicitly reported in paper (n=18)** | |
| --- | --- |
| **Author (year)** | **Definition** |
| Adu (2020) | System usage is measured through the collection of noninvasive data on the frequency of access to the app, push notifications openend, and average time spent per usage. |
| Aure (2021) | Total engagement is quantitatively defined as usage patterns (number of recording periods per day, number of total use days, and use days per week) and qualitatively defined as reflections on what contributed to or hindered regular use of Appetitus over time. |
| Baik (2020) | Total engagement was defined as use in mminutes per week throughout the study period. |
| Balk-Moller (2017) | Points are used as an indicator of total engagement. |
| Berman (2018) | Total engagement is defined as the average number of recorded app actions per day(eg, planning or reporting meals, scheduling calls, building shopping lists) |
| Bohm (2020) | User engagement was assessed as the number of active days and using measures expressing the persistence, longevity, and regularity of interaction within the first 180 days of use. |
| Buss (2024) | App engagement included active days, duration of app use, and frequency of accessing app modules. |
| Falkenhain (2022) | Engagement (1) Keyto  Levels obtained through use of the breath acetone biofeedback  device and (2) the number of engagements with the Keyto app |
| Huntiss (2024) | A day was counted as active if at least one of the following tracking activities was manually self‐reported: weight, physical activity, fasting (a fast was started or finished), food or drink. User in‐app activity is a proportion of active days in a specific observation period. The value of in‐app activity varies between 0.0% and 100.0%, where a higher value represents a higher engagement with the app. |
| Lim (2022) | App engagement was defined as actively using the individual app features. Table 1 describes definitions for engagement for all individual app features. |
| Lin (2018) | We defined engagement as the use of specific app components. For the PC arm only, we additionally defined engagement as attendance at group sessions and phone counseling calls. |
| Price (2020) | Total engagement is defined as the total number of times the app was engaged with all its functions |
| Serlachius (2019) | User engagement encompasses both how often and for how long people use apps as well as the user’s experience of the technology as a whole. In this study user engagement was measured using a modified user version te Mobile Application Rating Scale (uMARS) |
| Serrano (2017) | User engagement was operationalized as app use, that is, the number of days users logged in on the app. |
| Son (2023) | Engagement level was calculated by scoring the information obtained from the participant’s app usage data. To ensure equal weighting of the dietary coaching and smartband usage, the scores obtained from each were converted to a maximum of 50 points and then combined for a total out of 100. |
| Young (2021) | Effective engagement was examined by calculating pairwise correlations (Spearman Correlation) between metrics of use (Number of logins, Total time key activities, % videos watched, Number of food data entries, goal completion data, and % per protocol) and intensity of use engagement data (Food entries per week, Average time per login), and change in diet quality (change in MEDAS scores). |
| Young (2021) | Engagement was represented by duration, frequency, and intensity-of-use measures; 8 engagement measures were calculated from database entries, timestamped event logging from active sessions, and a custom script tracking the duration of videos watched. |

1. **List of included studies**
2. Adu MD, Malabu UH, Malau-Aduli AEO, Drovandi A, Malau-Aduli BS. User retention and engagement with a mobile app intervention to support self-management in Australians with type 1 or type 2 diabetes (my care hub): Mixed methods study. JMIR mHealth uHealth. 2020;8(6). doi:10.2196/17802
3. Ainscough KM, O’Brien EC, Lindsay KL, et al. Nutrition, behavior change and physical activity outcomes from the PEARS rct-an mhealth-supported, lifestyle intervention among pregnant women with overweight and obesity. 2019;10:938. doi:10.3389/fendo.2019.00938
4. Aure CF, Kluge A, Moen A. Older Adults’ Engagement in Technology-Mediated Self-Monitoring of Diet: A Mixed-Method Study. J Nurs Scholarsh. 2021;53(1):25-34. doi:10.1111/jnu.12619
5. Baik SH, Oswald LB, Buscemi J, et al. Patterns of use of smartphone-based interventions among latina breast cancer survivors: secondary analysis of a pilot randomized controlled trial. 2020;6(2). doi:10.2196/17538
6. Balk-Møller NC, Larsen TM, Holm L. Experiences from a web- And app-based workplace health promotion intervention among employees in the social and health care sector based on use-data and qualitative interviews. J Med Internet Res. 2017;19(10). doi:10.2196/JMIR.7278
7. Berman MA, Guthrie NL, Edwards KL, et al. Change in glycemic control with use of a digital therapeutic in adults with type 2 diabetes: Cohort study. JMIR Diabetes. 2018;20(2). doi:10.2196/diabetes.9591
8. Böhm AK, Jensen ML, Sørensen MR, Stargardt T. Real-world evidence of user engagement with mobile health for diabetes management: Longitudinal observational study. JMIR mHealth uHealth. 2020;8(11). doi:10.2196/22212
9. Brindal E, Hendrie G, Freyne J, Coombe M, Berkovsky S, Noakes M. Design and pilot results of a mobile phone weight-loss application for women starting a meal replacement programme. 2013;19(3):166‐174-. doi:10.1177/1357633X13479702
10. Brindal E, Hendrie GA, Freyne J, Noakes M. A Mobile Phone App Designed to Support Weight Loss Maintenance and Well-Being (MotiMate): randomized Controlled Trial. 2019;7(9):e12882-. doi:10.2196/1288210.
11. Buss VH, Barr M, Parker SM, et al. Mobile App Intervention of a Randomized Controlled Trial for Patients With Obesity and Those Who Are Overweight in General Practice: User Engagement Analysis Quantitative Study. JMIR mHealth uHealth. 2024;12(1). doi:10.2196/45942
12. Carpenter CA, Eastman A, Ross KM. Consistency With and Disengagement From Self-monitoring of Weight, Dietary Intake, and Physical Activity in a Technology-Based Weight Loss Program: Exploratory Study. JMIR Form  Res. 2022;6(2). doi:10.2196/33603
13. Carter MC, Burley VJ, Nykjaer C, Cade JE. Adherence to a smartphone application for weight loss compared to website and paper diary: Pilot randomized controlled trial. J Med Internet Res. 2013;15(4). doi:10.2196/jmir.2283
14. Chen YS, Wong JE, Ayob AF, Othman NE, Poh BK. Can Malaysian young adults report dietary intake using a food diary mobile application? A pilot study on acceptability and compliance. Nutrients. 2017;9(1). doi:10.3390/nu9010062
15. Chew HSJ, Chew NW, Loong SSE, et al. Effectiveness of an Artificial Intelligence-Assisted App for Improving Eating Behaviors: Mixed Methods Evaluation. J Med Internet Res. 2024;26:e46036-. doi:10.2196/46036
16. Chikwetu L, Daily S, Mortazavi BJ, Dunn J. Automated Diet Capture Using Voice Alerts and Speech Recognition on Smartphones: Pilot Usability and Acceptability Study. JMIR Form  Res. 2023;7. doi:10.2196/46659
17. Comulada WS, Swendeman D, Koussa MK, et al. Adherence to self-monitoring healthy lifestyle behaviours through mobile phone-based ecological momentary assessments and photographic food records over 6 months in mostly ethnic minority mothers. Public Health Nutr. 2018;21(4):679-688. doi:10.1017/S1368980017003044
18. Eisenhauer CM, Brito F, Kupzyk K, et al. Mobile health assisted self-monitoring is acceptable for supporting weight loss in rural men: a pragmatic randomized controlled feasibility trial. BMC Public Health. 2021;21(1). doi:10.1186/s12889-021-11618-7
19. Elbert SP, Dijkstra A, Oenema A. A Mobile Phone App Intervention Targeting Fruit and Vegetable Consumption: The Efficacy of Textual and Auditory Tailored Health Information Tested in a Randomized Controlled Trial. JOURNAL OF MEDICAL INTERNET RESEARCH. 2016;18(6). doi:10.2196/jmir.5056
20. Falkenhain K, Locke SR, Lowe DA, et al. Use of an mHealth Ketogenic Diet App Intervention and User Behaviors Associated With Weight Loss in Adults With Overweight or Obesity: Secondary Analysis of a Randomized Clinical Trial. JMIR mHealth uHealth. 2022;10(3). doi:10.2196/33940
21. Freyne J, Yin J, Brindal E, Hendrie GA, Berkovsky S, Noakes M. Push Notifications in Diet Apps: Influencing Engagement Times and Tasks. Int J Hum-Comput Interact. 2017;33(10):833-845. doi:10.1080/10447318.2017.1289725
22. Gilliland J, Sadler R, Clark A, O’Connor C, Milczarek M, Doherty S. Using a smartphone application to promote healthy dietary behaviours and local food consumption. BioMed Res Int. 2015;2015. doi:10.1155/2015/841368
23. Helander E, Kaipainen K, Korhonen I, Wansink B. Factors related to sustained use of a free mobile app for dietary self-monitoring with photography and peer feedback: Retrospective cohort study. J Med Internet Res. 2014;16(4). doi:10.2196/jmir.3084
24. Hendrie GA, Sazzad Hussain M, Brindal E, James-Martin G, Williams G, Crook A. Impact of a mobile phone app to increase vegetable consumption and variety in adults: Large-scale community cohort study. JMIR mHealth uHealth. 2020;8(4). doi:10.2196/14726
25. Henriksson P, Migueles JH, Söderström E, Sandborg J, Maddison R, Löf M. User engagement in relation to effectiveness of a digital lifestyle intervention (the HealthyMoms app) in pregnancy. Sci Rep. 2022;12(1). doi:10.1038/s41598-022-17554-9
26. Huntriss R, Salimgaraev R, Nikogosov D, Powell J, Varady KA. The effectiveness of mobile app usage in facilitating weight loss: An observational study. Obes Sci Pract. 2024;10(3). doi:10.1002/osp4.757
27. Kliemann N, Croker H, Johnson F, Beeken RJ. Development of the top tips habit-based weight loss app and preliminary indications of its usage, effectiveness, and acceptability: Mixed-methods pilot study. JMIR mHealth uHealth. 2019;7(5). doi:10.2196/12326
28. Kwon BC, VanDam C, Chiuve SE, et al. Improving heart disease risk through quality-focused diet logging: Pre-post study of a diet quality tracking app. JMIR mHealth uHealth. 2020;8(12). doi:10.2196/21733
29. Kytö M, Hotta S, Niinistö S, et al. Periodic mobile application (eMOM) with self-tracking of glucose and lifestyle improves treatment of diet-controlled gestational diabetes without human guidance: a randomized controlled trial. Am J Obstet Gynecol. Published online 2024. doi:10.1016/j.ajog.2024.02.303
30. Laing BY, Mangione CM, Tseng CH, et al. Effectiveness of a smartphone application for weight loss compared with usual care in overweight primary care patients: a randomized, controlled trial. 2014;161(10 Suppl):S5‐12-. doi:10.7326/M13-3005
31. Lee J, Bae S, Park D, Kim Y, Park J. The effectiveness of a monetary reimbursement model for weight reduction via a smartphone application: a preliminary retrospective study. Sci Rep. 2020;10(1). doi:10.1038/s41598-020-72908-5
32. Lim SL, Juan Tay MH, Ong KW, et al. Association Between Mobile Health App Engagement and Weight Loss and Glycemic Control in Adults With Type 2 Diabetes and Prediabetes (D’LITE Study): Prospective Cohort Study. JMIR Diabetes. 2022;7(3). doi:10.2196/35039
33. Lin PH, Grambow S, Intille S, et al. The association between engagement and weight loss through personal coaching and cell phone interventions in young adults: Randomized controlled trial. JMIR mHealth uHealth. 2018;6(10). doi:10.2196/10471
34. Moyen A, Rappaport AI, Fleurent-Grégoire C, Tessier AJ, Brazeau AS, Chevalier S. Relative Validation of an Artificial Intelligence-Enhanced, Image-Assisted Mobile App for Dietary Assessment in Adults: Randomized Crossover Study. J Med Internet Res. 2022;24(11). doi:10.2196/40449
35. Mummah S, Robinson TN, Mathur M, Farzinkhou S, Sutton S, Gardner CD. Effect of a mobile app intervention on vegetable consumption in overweight adults: A randomized controlled trial. Int J Behav Nutr Phys Act. 2017;14(1). doi:10.1186/s12966-017-0563-2
36. Mummah SA, Mathur M, King AC, Gardner CD, Sutton S. Mobile technology for vegetable consumption: A randomized controlled pilot study in overweight adults. JMIR mHealth uHealth. 2016;4(2). doi:10.2196/mhealth.5146
37. Nezami BT, Hurley L, Power J, Valle CG, Tate DF. A pilot randomized trial of simplified versus standard calorie dietary self-monitoring in a mobile weight loss intervention. Obesity. 2022;30(3):628-638. doi:10.1002/oby.23377
38. Pagoto S, Tulu B, Agu E, Waring ME, Oleski JL, Jake-Schoffman DE. Using the habit app for weight loss problem solving: Development and feasibility study. JMIR mHealth uHealth. 2018;6(6). doi:10.2196/mhealth.9801
39. Pagoto S, Tulu B, Waring ME, et al. Slip buddy app for weight management: Randomized feasibility trial of a dietary lapse tracking app. JMIR mHealth uHealth. 2021;9(4). doi:10.2196/24249
40. Patel ML, Hopkins CM, Brooks TL, Bennett GG. Comparing self-monitoring strategies for weight loss in a smartphone app: Randomized controlled trial. JMIR mHealth uHealth. 2019;7(2). doi:10.2196/12209
41. Payne JE, Turk MT, Kalarchian MA, Pellegrini CA. Adherence to mobile-app-based dietary self-monitoring—Impact on weight loss in adults. Obes Sci Pract. 2022;8(3):279-288. doi:10.1002/osp4.566
42. Prapkree L, Sadjadi M, Huffman F, Palacios C. Development and pilot testing of the snackability smartphone application to identify healthy and unhealthy snacks. Healthc Informatics Res. 2019;25(3):161-172. doi:10.4258/hir.2019.25.3.161
43. Price M, Higgs S, Wilkinson L, et al. Construal beliefs moderate the usability and effectiveness of a novel healthy eating mobile app. Physiol Behav. 2020;222. doi:10.1016/j.physbeh.2020.112941
44. Riches SP, Piernas C, Aveyard P, et al. A mobile health salt reduction intervention for people with hypertension: Results of a feasibility randomized controlled trial. JMIR mHealth uHealth. 2021;9(10). doi:10.2196/26233
45. Robinson E, Higgs S, Daley AJ, et al. Development and feasibility testing of a smart phone based attentive eating intervention. BMC Public Health. 2013;13(1). doi:10.1186/1471-2458-13-639
46. Ruf A, Koch ED, Ebner-Priemer U, Knopf M, Reif A, Matura S. Studying microtemporal, within-person processes of diet, physical activity, and related factors using the appetite-mobile-app: feasibility, usability, and validation study. 2021;23(7):e25850-. doi:10.2196/25850
47. Serlachius A, Schache K, Kieser A, Arroll B, Petrie K, Dalbeth N. Association between user engagement of a mobile health app for gout and improvements in self-care behaviors: Randomized controlled trial. JMIR mHealth uHealth. 2019;7(8). doi:10.2196/15021
48. Serrano KJ, Coa KI, Yu M, Wolff-Hughes DL, Atienza AA. Characterizing user engagement with health app data: a data mining approach. Transl Behav Med. 2017;7(2):277-285. doi:10.1007/s13142-017-0508-y
49. Simpson A, Gemming L, Baker D, Braakhuis A. Do image-assisted mobile applications improve dietary habits, knowledge, and behaviours in elite athletes? A pilot study. Sports. 2017;5(3). doi:10.3390/sports5030060
50. Son I, Hong J, Han YH, et al. Effectiveness of a mobile health intervention on weight loss and dietary behavior changes among employees with overweight and obesity: a 12-week intervention study investigating the role of engagement. Korean J Community Nutr. 2023;28(2):141-159. doi:10.5720/kjcn.2023.28.2.141
51. Spring B, Pellegrini C, McFadden HG, et al. Multicomponent mHealth intervention for large, sustained change in multiple diet and activity risk behaviors: The make better choices 2 randomized controlled trial. J Med Internet Res. 2018;20(6). doi:10.2196/10528
52. Springer A, Venkatakrishnan A, Mohan S, Nelson L, Silva M, Pirolli P. Leveraging self-affirmation to improve behavior change: A mobile health app experiment. JMIR mHealth uHealth. 2018;6(7). doi:10.2196/mhealth.9151
53. Su Y, Wu KC, Chien SY, Naik A, Zaslavsky O. A Mobile Intervention Designed Specifically for Older Adults With Frailty to Support Healthy Eating: Pilot Randomized Controlled Trial. JMIR Form  Res. 2023;7(1). doi:10.2196/50870
54. Surendran S, Lim CS, Koh GCH, Yew TW, Tai ES, Foong PS. Women’s usage behavior and perceived usefulness with using a mobile health application for gestational diabetes mellitus: Mixed-methods study. Int J Environ Res Public Health. 2021;18(12). doi:10.3390/ijerph18126670
55. Tay I, Garland S, Gorelik A, Wark JD. Development and testing of a mobile phone app for self-monitoring of calcium intake in young women. JMIR mHealth uHealth. 2017;5(3). doi:10.2196/mhealth.5717
56. Teong LF, Khor BH, Purba KR, et al. A Mobile App for Triangulating Strategies in Phosphate Education Targeting Patients with Chronic Kidney Disease in Malaysia: Development, Validation, and Patient Acceptance. Healthcare (Basel). 2022;10(3). doi:10.3390/healthcare10030535
57. Torbjørnsen A, Jenum AK, Småstuen MC, et al. A low-intensity mobile health intervention with and without health counseling for persons with type 2 diabetes, part 1: Baseline and short-term results from a randomized controlled trial in the norwegian part of RENEWING HEALTH. JMIR mHealth uHealth. 2014;2(4). doi:10.2196/mhealth.3535
58. Whitelock V, Kersbergen I, Higgs S, Aveyard P, Halford JCG, Robinson E. User experiences of a smartphone-based attentive eating app and their association with diet and weight loss outcomes: Thematic and exploratory analyses from a randomized controlled trial. JMIR mHealth uHealth. 2020;8(10). doi:10.2196/16780
59. Young CL, Mohebbi M, Staudacher H, Berk M, Jacka FN, O’Neil A. Assessing the feasibility of an m-Health intervention for changing diet quality and mood in individuals with depression: the My Food & Mood program. Int Rev Psychiatry. 2021;33(3):266-279. doi:10.1080/09540261.2020.1854193
60. Young CL, Mohebbi M, Staudacher HM, et al. Optimizing engagement in an online dietary intervention for depression (My food & mood version 3.0): Cohort study. JMIR Ment Heal. 2021;8(3). doi:10.2196/24871
61. **Basic study characteristics**

|  |  | **STUDY CHARACTERISTICS: POPULATION** | | | | | | | |
| --- | --- | --- | --- | --- | --- | --- | --- | --- | --- |
| **Author** | **Year** | **Study Design** | **Location** | **Total No participants** | **Female/Male** | **Duration** | **Age(SD)** | **BMI(kg/m2)(SD)** | **Condition/Disease** |
| Adu | 2020 | Explanatory mixed methods design | Australia | 50 (42 adhered to study protocol) | 19/31 (baseline) and 25/16 (completers) | 3 weeks | 49.29 (12.74) | Not reported. | Patients with type 1 or type 2 diabetes, having a current recommended blood glucose target of 4 to 10 mmol/L |
| Ainscough | 2019 | RCT | Ireland | 565 (intervention: 278, control: 287) | 565 (no males) | Data was collected within 2 years. | Intervention: 32.84 (4.60). Control: 32.33 (4.23) | 29.44 (SD: 3.60) | Pregnant women (10–18 weeks' gestation) with overweight and obesity (BMI ≥25 kg/m2 and ≤39.9 kg/m2) |
| Aure | 2021 | Explorative mixed methods | Norway | 25 | 18 female, 7 male | 8 weeks | 79.48 (no SD, but range: 68 - 95) | Not reported. | Older adults with good cognitive health. |
| Baik | 2020 | Secondary Analysis of a Pilot RCT | United States | 78 | N.a. | 6 weeks (2 hours per week) | 52.54 (11.36) | Not reported. | Women diagnosed with nonmetastatic stage 0-III breast cancer, who had completed active treatment for breast cancer (within 2-23 months of completing treatment) |
| Balk-Møller | 2017 | Mixed-methods design (secondary analysis of RCT + interviews & focus groups) | Denmark | RCT: 556, Qualitative: 38 | RCT; intervention: 140/12, control: 108/9. Qualitative: not reported. | RCT; 38 weeks. Qualitative; not reported | 47 (SD; intervention: 10, control: 9.9) | Mean height: 166.2 cm (SD: 7, intervention), 165.5 cm (SD 7.2, control). Mean weight: 74.5 kg (SD: 15.9, intervention), 73.1 (SD: 14.8, control). | Not reported. |
| Berman | 2018 | Non-blinded, single-arm interventional study | USA | 118 | 96/22 | 12 weeks | 50.7(9.4) | 38.1 (8.8) | self-reported HbA1c> 6.5% |
| Böhm | 2020 | Longitudinal Observational Study | US patients | 9051 | Among users who shared information about their gender, 56.86% (F:2075/M:1574 out of 3649) reported to be female. It should be noted that only 40.32% (3649/9051) of all users provided information on their gender | 180 days | 50.4(13.5) | 34.83 (9.79) | 63.50% (5747/9051) of the users reported being diagnosed with T2D and 13.48% (1220/9051) with T1D. |
| Brindal | 2019 | RCT | Australia | 88 (61 completed it) | 66/22 | 24 weeks | 45.13 (13.19)/ I:44.5 (13.39)/ C:45.8 (13.11) | between 20.9 and 60.8 kg/m2 . | normal weight, overweight, obese |
| Brindal | 2013 | pilot RCT | Australia | 58 | 58 (only women) | 8 weeks | 42 | 26–43 kg/m2 (mean 34) | overweight/obese |
| Buss | 2024 | Subanalysis/secondary analysis of Cluster RCT | Australia | 120 | 60/60 | 6 weeks, follow up health review after 12-weeks | Nonapp users: 58 (SD: 8), app users (61 (SD: 9) | Not reported | BMI of ≥28 |
| Carpenter | 2022 | pilot RCT | USA | 54 | 47/7 | 6 months | 49.6(12.4) | 32.6 (3.2) | overweight/obese |
| Carter | 2013 | pilot RCT | UK | 128 | 99/29 | 6 months | 42(9) | 34(5) | overweight/obese |
| Chen | 2017 | Mixed Methods Evaluation | Malaysia | 28 | 25/3 | NA | 22() | 20.1 (2.3) | healthy university students |
| Chew | 2024 | Mixed Methods Evaluation, single-group pretest-posttest | Singapore | 251 (1 removed from analysis due to ineligibility) | 131/119 | 12 weeks | Completed: 31.25 (9.98), dropped out: 36.23 (10.29) | Completed: 28.86 (7.02), dropped out: 33.73 (8.21) | Participants older than 21 years with BMI ≥23 kg/m2 and not undergoing a commercial weight loss program |
| Chikweth | 2023 | 2-arm study | USA | 18 | 8//10 | 28 days | 20-39 years | Not reported. | Not reported. |
| Comulada | 2018 | controlled trial (but the control was not implemented to | USA | 42 | 42/0 | 6 months | 20-43 years | 32.4 (7) | mothers |
| Eisenhauer | 2021 | RCT feasibility trial | USA | 80 | 0/80 | 6 months | 54.2(8.6) | 35.6(6.9) | overweight and obese |
| Elbert | 2016 | RCT | Netherlands | 146 | 107/45 | 6 months | 41.4(14.6) | 25.2(5.5) | Not specified. |
| Falkenhain | 2022 | RCT | USA | 75 | 53/20/2 (n=2 non binary) | 24 weeks | 42(11) | 33.5(4.7) | overweight/obese |
| Freyne | 2017 | efficacy trial | Australia | 75 | 55/20 | 24 weeks | 48.57 | not reported | overweight/obese |
| Gilliland | 2015 | Single-arm uncontrolled prospective study | Canada | 208 | Not reported. | 8-10 weeks | 33 | not reported | Not reported. |
| Helander | 2014 | Retrospective Cohort Study | N/A | 189770 | Not reported. | N/a | not reported | not reported | not reported |
| Hendrie | 2020 | Large-Scale Community Cohort Study | Australia | 5062 | 4265/774/23(unisex) | 90 days | 48.2(14.1) | not reported | not reported |
| Henriksson | 2022 | secondary analysis of a Randomized Controlled Trial | Sweden | 305 | 305/0 | 6 months | only data from 134 women in the intervention group are shown: 31.5(4.2) | only data from 134 women in the intervention group and from 37 gestational week: 28.5(4.2) | pregnant women |
| Huntriss | 2024 | Analysis of data from SIMPLE, a free mobile application with several features available only in a paid subscription plan. | USA | 53.482 | 12 weeks: 26,690/10,260, 26 weeks: 15,667/6423, 52 weeks: 9791/4449 | N/a: Three observation periods: 12, 26, of 52 weeks. | 12 weeks: 43.10 (11.79); 26 weeks: 43.17 (11.72); 52 weeks: 43.56 (11.55) | Baseline BMI (median, IQR): 12 weeks: 31 (27.93, 35.42); 26 weeks: 31 (27.89, 35.24), 52 weeks: 31 (27.73, 34.88) | SIMPLE application users were included, excluding those who were pregnant, breastfeeding, or reported an eating disorder |
| Kliemann | 2019 | Mixed-Methods Pilot Study | UK | 81 | 73/8 | 3 months | 42.4(13.4) | 34.3(7) | overweight/obese |
| Kwon | 2020 | Pre-Post Study | US | 32 | 26/6 | 5 weeks | 57.5(11.9) | not reported | overweight or obese with high heart disease risk |
| Kyto | 2024 | RCT | Helsinki, Finland | 148 | 148/0 | 24-28 weeks of pregnancy till 35-37 weeks of pregnancy (not specified duration) | C; 48.6 (35)/I: 48.7 (37) | C:26.7(1.1)/I: 27.5(1.3) | pregnancy |
| Laing | 2014 | RCT | USA, Los Angeles | 212 | 154/58 | 6 months | 43.3(14.3) | 33.4(7.1) | overweight ot obese |
| Lee | 2020 | a preliminary retrospective study | Korea | 2803 | 2439/364 | 16 weeks | 33.6(7.9) | Mission success, n=1564, BMI: 24.1(2.7)/ Mission failure, n=1238, BMI: 24.4(2.8) | overweight or obese |
| Lim | 2022 | Prospective Cohort Study | Singapore | 171 | 109/62 | 6 months | 52 | 29.3 | Diabetes and prediabetes |
| Lin | 2018 | RCT(but in that paper they only analyse data from the interventions) | USA | 242 | 169/73 | 24 weeks | 29.3(4.2) | 35.3(7.9) | overweight or obese |
| Moyen | 2022 | Randomized Crossover Study | Canada | 136 | 87/49 | 2 weeks | 46.1(14.6) | women: 24(3.9)/ men: 26.8(4.4) | not specified; inclusion criteria a self-reported BMI between18 and 35 kg/m2 |
| Mummah | 2016 | Pilot RCT | USA | 17 | F:11/ M: 6 | 12 weeks | 42(7.3) | 32(3.5) | overweight |
| Mummah | 2017 | RCT | USA | 135 | 84/51 | 12 months | Control: 40.3(5.8)/ Intervention: 39.4(6.7) | 28–40 kg/m2 | overweight |
| Nezami | 2022 | Pilot RCT | USA, North Carolina | 72 | Standard: 35/2, Simplified: 33/2 | 6 months | Standard: 39.8 (4.7), Simplified: 40.2 (4.7) | Standard: 35.3 (SD: 6.8), Simplified: 33.07 (SD: 5.7) | Overweight/obese |
| Pagoto | 2018 | Two iterative single-arm pilot studies | USA, Connecticut | 43 (Pilot 1: 27, Pilot 2: 16) | Pilot 1: 18/9, Pilot 2: 12/4 | Both studies lasted 8 weeks | Pilot 1: 37.22 (11.55), Pilot 2: 37.35 (10.85) | Pilot 1: 31.14 (SD: 4.63), Pilot 2: 32.96 (SD: 5.99) | Overweight/obese |
| Pagoto | 2021 | RCT | USA, Connecticut | 64 | Slip Buddy: 24/8, Calorie Tracking: 24/8 | 12 weeks | Slip Buddy: 39.5 (9.6), Calorie Tracking: 40.2 (12.3) | Slip Buddy: 34.9 (SD: 5.3), Calorie Tracking: 33.4 (SD: 4.4) | Overweight/obese adults |
| Patel | 2019 | RCT | USA, North Carolina | 105 | 84/16 | 12 weeks | 42.7 (11.7) | 31.9 (SD: 4.5) | Overweight or obese |
| Payne | 2022 | Single-arm uncontrolled prospective study | USA, North Carolina | 90 | 87/3 | 8 weeks | 42.8 (10.1) | 35.1 (SD: 6) | Overweight or obese |
| Prapkree | 2019 | Mixed-methods design | USA, Florida | 20 (12 for the first pilot and 8 for the second pilot) | Not reported. | 2 weeks for pilot 1 and 2 weeks for pilot 2 | No mean reported. Range: 18-24. | Not reported. | Not reported. |
| Price | 2020 | Iterative mixed-methods design | UK | 71 | 51/20 | Development: 18 months Longitudunal trial: 8 weeks | 33.34 (11.68) | 26.22 (SD: 4.94) | Not reported. |
| Riches | 2021 | Feasibility Randomized Controlled Trial | UK, Oxford | 47 | 30/17. Control: 10/6, Intervention: 20/11 | 6 weeks | 65 (11) | 29 (6) | Adults with a recent blood pressure reading) |
| Robinson | 2013 | Feasibility trial | UK, Birmingham | 12 | 7 females/5 males | 4 weeks | 41.7 (12.4) | 32.1 (SD: 5.3) | Overweight/obese |
| Ruf | 2021 | (Longitudinal) crossover study design | Germany | 157 | 100/57 | 7 days | 28.04 (7.22) | 24.71 (SD: 4.81) | individuals who were not affected by psychiatric conditions, patients affected by attention-deficit/hyperactivity disorder, patients with an increased risk for the development of bipolar disorder, including patients affected by attention-deficit/hyperactivity disorder or depression. A sample combining healthy and psychiatric patients. |
| Serlachius | 2019 | RCT | New Zealand, Auckland | 72 | Gout Central: 6/30, DASH diet: 32/4 | 2 weeks | Gout Central: 45 (14); DASH Diet: 53 (15) | Not reported. | Adults with a diagnosis of gout as defined by the 2015 ACR-EULAR Gout Classification Criteria |
| Serrano | 2017 | Secondary data analysis of cross-sectional data | USA, Maryland | 1,011,008 | Subgroup 1: 76.6% female, Subgroup 2: 74.7% female, Subgroup 3: 74.2% female, Subgroup 4: 78.9% female, Subgroup 5: 71.3% female, and Subgroup 6: 75.8% female. | N.a. | Subgroup 1: 34.3 (12.7), Subgroup 2: 35.1 (12.2), Subgroup 3: 37.1 (12.5), Subgroup 4: 36.5 (12.3), Subgroup 5: 36.7 (11.9), and Subgroup 6P 36.8 (11.7) | Subgroup 1: 30.0 (SD: 7.3), Subgroup 2: 30.8 (SD: 7.2), Subgroup 3: 30.8 (SD: 7.1), Subgroup 4: 29.7 (SD 6.6), Subgroup 5: 30.6 (SD: 7.2), and Subgroup 6: 29.8 (SD: 6.7) | Users of a weight loss app |
| Simpson | 2017 | Pilot feasibility trial | New Zealand, Auckland | 17 | 0/17 | 6 weeks | 19 (0.7) | Not reported. | Elite male field hockey players (athletes) |
| Son | 2023 | non controlled, single arm study | Korea | 235 | 51/184 | 12 weeks | 36.9 ± 8.7 | 27.5 ± 63.3 | overweight or obese |
| Spring | 2018 | RCT | USA, Chicago | 212 | 162/50 | 9 months | 40.8 (11.9) | 34.3 (SD: 8.8) | Adults of 18-65 years consuming less than 5 servings of fruits and vegetables per day; ≥8% daily calories from saturated fat; <150 minutes per week MVPA; >120 minutes per week of leisure screen time |
| Springer | 2018 | 2×2 factorial design | USA, California | 127 | 90/36, and 1 identified as non-binary | 28 days | Not reported. | Not reported. | Adults with fruit and vegetable consumption below recommended levels |
| Surendran | 2021 | Mixed-methods study: secondary analysis of a RCT and Semi-structured interviews | Singapore | RCT: 170 (intervention arm), Qualitative: 14 | RCT: 170/0, Qualitative: 14/0 | 8 weeks | RCT; range: 24-32 (average: 32). Qualitative; range: 27-36 (average: 36). | Not reported. | Women diagnosed with gestational diabetes mellitus between 12 and 30 weeks of gestation. |
| Su | 2023 | pilot RCT | USA | 15 | 14;1 | 3months | 66-77 (mean 70.5, SD 3.96) years | I: 26.88(6.86)/ C: 29.04(4.39) | older adults with frailty, |
| Tay | 2017 | Development and mixed methods evaluation (usability and acceptability) | Australia, Parkville | 40 | 40/0 | 5 days (over 2 weeks) | Not reported. | Not reported. | Healthy females of 16-25 years old |
| Teong | 2022 | Development, validation, and acceptability study (cross-sectional study) | Malaysia | Expert validation: n = 5, Evalatuion of app acceptance: n = 139 | Expert validation: not reported. Evaluation of app acceptance: 69/70 | N.a. | Expert validation: not reported. Validation: 48.1 (13.2) | Not reported. | Experts who were nephrologists (n = 5), renal pharmacists (n = 2), and senior dietitians (n = 6) from both public (n = 11) and private (n = 2) institutions. In the validation study, stable hemodialysis patients were included. |
| Torbjørnsen | 2014 | RCT | Norway | 151 | 62/89 | 1 year | 57 (12) | 31.7 (SD: 6.0) | Adults with type 2 diabetes and HbA1c ≥7.1% |
| Whitelock | 2020 | Thematic and Exploratory Analyses from a RCT | UK. Liverpool | 39 | Female: 31; Male: 8 | 8 weeks | 41.7 (10.3) | 35.2 (SD: 7.2) | Adults with overweight and obesity (mean BMI ≥25 kg/m2) |
| Young | 2021 | Single arm cohort | Australia, Victoria | 153 | 125/28 | 8 weeks | 42.4 (median q1, g3: 33, 50) | 26 (SD: 6) | Adults with depressive symptoms (score of >5 on PHQ-8). |
| Young | 2021 | Single arm cohort design | Australia, Victoria | 614 | 536/78 | 8 weeks | Cohort 1: 40 (median q1, q3: 32, 49); Cohort 2.1: 37 (median q1, q3: 30, 45); Cohort 2.2: 41 (median q1, q3: 34, 49); Cohort 3: 42.5 (median q1, q3: 33, 50). | Cohort 1: 26.41 (SD: 5.68); Cohort 2.1: 25.85 (SD: 7.19); Cohort 2.2: 26.09 (SD: 6.05); Cohort 3: 26.28 (SD: 6.11). | Adults with depressive symptoms (score of >5 on PHQ-8). |

1. **Table with the characteristics of the apps**

| **Author** | **Source** | **Engagement strategies** | **Evaluation** |
| --- | --- | --- | --- |
| Adu | Research | Behavioral theory, push notifications & prompts | NR |
| Ainscough | Research | Not specifically mentioned, but 'daily app usage was encouraged', hinting towards prompts? But it could, Behavioral theory was used to inform, but was not specifically targeted at, but provision of a daily exercise | NR |
| Aure | Research | Peronsalization, customization, Perhaps also goal setting, presented with a mealplan to meat | feasibility |
| Baik | Research | Access to professionals, multimedia formats, behavioral theory, goal setting | feasibility |
| Balk-Møller | Research | Personalization, customization, goal setting, game like features, peer to peer communication | NR |
| Berman | Research | Bhevaioral theory | satisfaction |
| Böhm | Commercial | Real time Data Monitoring, Passive Monitoring |  |
| Brindal | Reseach | Push Notifications, Prompts | satisfaction |
| Brindal | Commercial | Push Notifications, Prompts | satisfaction |
| Buss | Research | Goal setting, access to health professional | Not mentioned |
| Carpenter | Commercial | Real time data monitoring, push notifications ;prompts | Not mentioned |
| Carter | research | None of categories, but tailored weekly text messages | acceptability |
| Chen | Research | No user engagement strategies | acceptability |
| Chew | Research | Behavioral theory, personalization, customization, prompts | satisfaction |
| Chikweth | Research | prompts, personalised alerts | usability |
| Comulada | Research | prompts | Daily app use |
| Eisenhauer | Research | self monitoring of, activity, along with personalized reporting, goal setting | Daily app use |
| Elbert | Research | Personalization, customization, behavior change theory, push notifications;prompts | applicability, novelty, credibility |
| Falkenhain | Commercial | NR | NR |
| Freyne | Research | notifications, prompts, game like features | Satisfaction |
| Gilliland | Research | Push notifications, behavioral theory | Satisfaction |
| Helander | Commercial | nan | NR |
| Hendrie | Commercial | Feedback, motivational messages, Challenges, Awads, personalization & customizations | NR |
| Henriksson | Research | push notofications, recipes, library, pregnancy calendar, slef monitoring features, information themes | NR |
| Huntriss | Commercial | SIMPLE app provides trackers for fasting, food, drinks, physical activity, weight, option to integrate data from third‐party | NR |
| Kliemann | Research | Habit theory | Acceptability |
| Kwon | Research | behavioral change techniques | NR |
| Kyto | Research | not mentioned | not mentioned |
| Laing | Commercial | behavioral change tecnhiques for weight loss, social networking | satisfaction |
| Lee | Commercial | technology enhanced features, usability, incorporations of behavior change technique | NR |
| Lim | Research | behavioral science, prompts, alerts, chat function | NR |
| Lin | Research | behavioral change strategies | NR |
| Moyen | Research | Push notifications, prompts | Usability |
| Mummah | Research | behavioral change theory | usability , satisfaction |
| Mummah | Research | game liked features, behavioral change, challenges, prompts | satisfaction |
| Nezami | Research | Push notifications, prompts, personalization, customization, goal setting, real time data monitoring | Satisfaction, acceptability |
| Pagoto | Research | Push notifications, prompts, personalization & customization, goal setting, multi media formats, real time data monitoring | Usability, acceptability, feasibility |
| Pagoto | Research | Push notifications, prompts, personalization, customization, goal setting, peer to peer communication | Usability, acceptability, feasibility |
| Patel | Commercial | Push notifications, prompts, goal setting, behavioral theory | NR |
| Payne | Commercial | Push notifications, prompts, goal setting, behavioral theory | NR |
| Prapkree | Research | Game like features, personalization, customization | Feasibility ,usability , satisfaction, acceptability |
| Price | Research | Personalization, customization, prompts, cues | Feasibility, usability |
| Riches | Research | Personalizatoin, customizatoin, goal setting, behavioral theory | feasibility |
| Robinson | Research | Push Notifications, Prompts, Personalization, Customization, Behavioral Theory | feasibility |
| Ruf | Research | Push notifications, prompts | Feasibility, usability |
| Serlachius | Commercial | Personalization, customization | NR |
| Serrano | Commercial | Personalization, customization, goal setting, peer to peer communication, push notification, prompts | NR |
| Simpson | Commercial | Customization, pesonalization, peer to peer communication | Feasibility, usability |
| Son | Commercial | encouraging messages, phone calls, weekly events with awards |  |
| Spring | Research | Goal setting | NR |
| Springer | Research | Push notifications, prompts, goal setting | NR |
| Surendran | Research | Prompts, push notifications, real time data monitoring, passive monitoring, access to professionals, behavioral theory | NR |
| Su | research | shaping knowledge, goal setting, feedback, reminders, Positive Outcome Expectations, Negative Outcome Expectations | feasibility , acceptability |
| Tay | Research | Push notifications, promps | Acceptability . usability |
| Teong | Research | Push notifications, prompts, goal setting | acceptability |
| Torbjørnsen | Research | Goal setting, access to professionals, behavioral theory | NR |
| Whitelock | Research | Game like features, multimedia formats | feasibility |
| Young | Research | Push notifications, prompts, goal setting, multimedia formats, access to professionals, behavioral theory | feasibility |
| Young | Research | Goal setting, game like features, multimedia formats | NR |

Figure 2A. Study characteristics and app focus of included nutrition apps (n = 59).


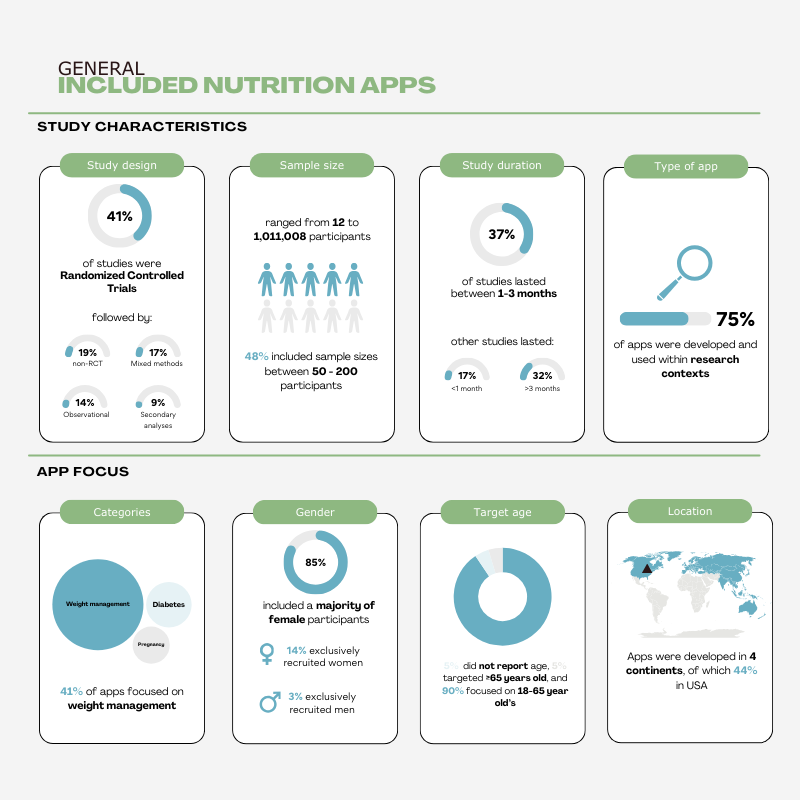


Figure 3A. User engagement strategies and metrics used in included nutrition apps (n = 59). The top panel shows the types of user engagement strategies identified across included studies, expressed as the percentage in which they were used. The light blue shading and star indicate the most used strategies (top three). The bottom panel presents the frequency of assessed user metrics and the percentage of apps that reported each.


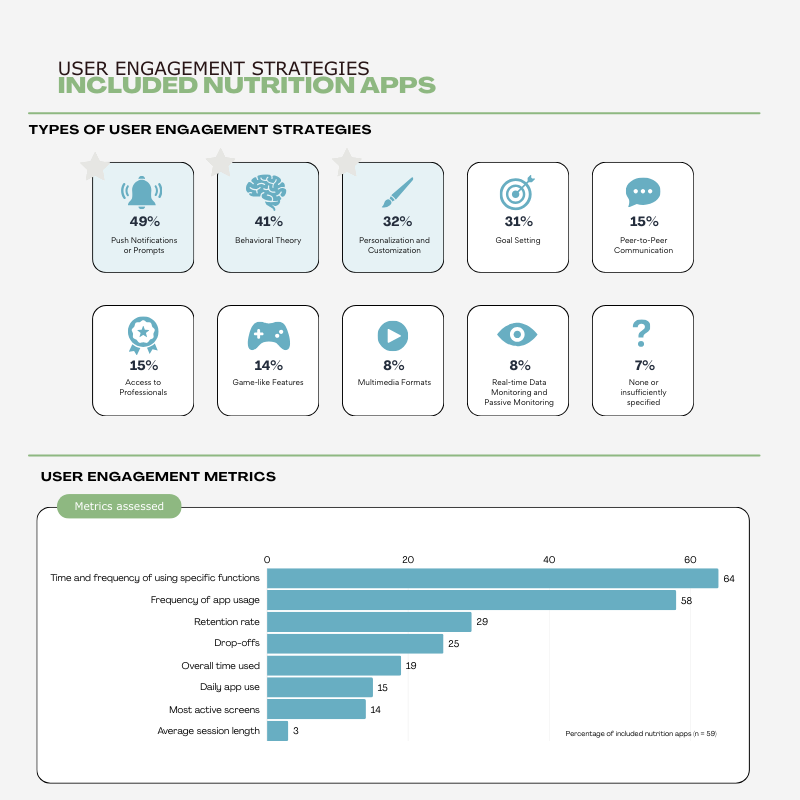

Supplement: Multimedia Appendix 1 [file mhealth_v14i1e82276_app1.docx]
